# Absorption and Metabolism of Urolithin A and Ellagic Acid in Mice and Their Cytotoxicity in Human Colorectal Cancer Cells

**DOI:** 10.1155/2023/8264716

**Published:** 2023-09-05

**Authors:** I-Chen Lin, Jin-Yi Wu, Chuan-Yin Fang, Shou-Chie Wang, Yi-Wen Liu, Shang-Tse Ho

**Affiliations:** ^1^Department of Colon-Rectal Surgery, Ditmanson Medical Foundation Chiayi Christian Hospital, Chiayi 600, Taiwan; ^2^Department of Microbiology, Immunology and Biopharmaceuticals, College of Life Sciences, National Chiayi University, Chiayi 600, Taiwan; ^3^Division of Nephrology, Department of Internal Medicine, Kuang Tien General Hospital, Taichung 437, Taiwan; ^4^Department of Wood Based Materials and Design, College of Agriculture, National Chiayi University, Chiayi 600, Taiwan

## Abstract

**Background:**

Ellagic acid is a natural polyphenol compound found in pomegranates, walnuts, and many berries. It is not easily absorbed, but it could be metabolized to urolithins by the gut microbiota. Urolithin A, one of the ellagic acid metabolites, has been proved to prolong the lifespan of *C. elegans* and increases muscle function of mice. The purpose of this current study was to analyze the absorption and metabolites of urolithin A and ellagic acid in mice and the anticancer effects of urolithin A, urolithin B, and ellagic acid in colorectal cancer cells.

**Methods:**

Urolithin A and urolithin B were synthesized and analyzed by HPLC and NMR. A pharmacokinetic study of urolithin A was performed in mice by analyzing urolithin A and its metabolites in urines. Absorption and biotransformation of ellagic acid were also studied in mice by analyzing the plasma, liver, and feces. The cytotoxicity of urolithin A, urolithin B, and ellagic acid was assayed in SW480, SW620, HCT 116, and HT-29 cells.

**Results:**

Urolithin A and urolithin B were synthesized and purified to reach 98.1% and 99% purity, respectively, and the structures were identified by NMR. In urolithin A intake analysis, urolithin A was only detectable at 3 h, not at 6–24 h; it suggested that urolithin A was rapidly metabolized to some unknown metabolites. Using UPLC-MS/MS analysis, the metabolites might be urolithin A 3-O-glucuronide, urolithin A 3-sulfate, and urolithin A-sulfate glucuronide. After feeding mice with ellagic acid for consecutive 14 days, ellagic acid contents could be detected in the fecal samples, but not in plasma and liver, and urolithin A was not detected in all samples. It suggests that ellagic acid is not easily absorbed and that the biotransformation of ellagic acid to urolithin A by intestinal flora might be very low. From the cytotoxicity assay, it was found that there was anticancer effect in urolithin A and urolithin B but not in ellagic acid. In contrast, ellagic acid promoted the proliferation of SW480 and SW620 cells.

## 1. Introduction

**Figure 1:**
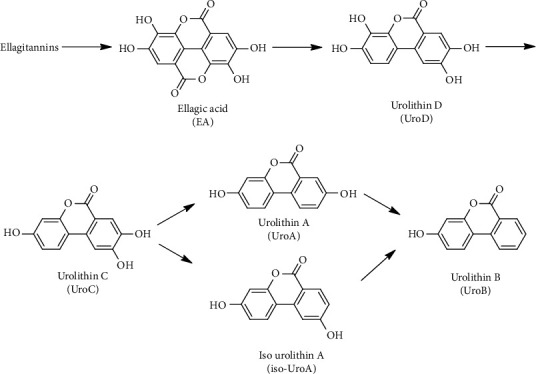
The metabolic pathway of ellagitannins and ellagic acid to urolithins.

Colorectal cancer (CRC) is one of the most common malignant tumors and the second leading cause of cancer-related deaths worldwide [1]. Early diagnosis and promotion of healthy eating habits have been established as basic preventive measures for CRC prevention [2]. Both hereditary and environmental risk factors play a role in the development of colorectal cancer [3]. In terms of environmental risk factors, an aging population and dietary habits are the most common factors [3]. Epidemiological studies have shown that the incidence of CRC is inversely related to the large intake of fruits and vegetables rich in phytochemicals, including phenolic compounds [4]. However, although it has been reported that different single phenols and phenol-rich plant foods have rich preclinical (in vitro and animal model) anticancer activity, so far, clinical evidence is still elusive [5]. Ellagic acid, which can be released from ellagitannins, is a polyphenol found in pomegranates, walnuts, and various berries. Ellagitannins, also known as hydrolysable tannins, are not absorbed in the gastrointestinal tract because they adhere to the Lipinskin rule of five [5]. Ellagic acid exhibits low bioavailability, with a maximum concentration of 120 ng/ml in human plasma [6]. It is known that ellagic acid is metabolized by the colonic flora and colorectal cells to produce a family of bioavailable metabolites called urolithins, which are 25∼80-fold more bioavailable than ellagic acid [6]. Remarkably, urolithins reach relevant concentrations in the lumen and colonic tissues (malignant and normal) of CRC patients after the intake of an ellagic acid-rich pomegranate extract [7]. Urolithin D is produced from ellagic acid through the opening of a lactone ring and the removal of a carboxyl group. Urolithin D loses 1, 2, or 3 hydroxyl groups through dehydroxylation to form urolithin C, urolithin A/isourolithin A, and urolithin B, respectively [6] (Figure 1). According to the constitution of urolithins in urine, the gut microbiota composition in humans differentially determines the 3 urolithin metabotypes including A, B, and 0 [8]. This dis-similar metabolism is linked to the interindividual variability of the human gut microbiota, which has led to identify three major final urolithins, including urolithin A, urolithin B, and isourolithin A [9].

Gut microbiota plays an important role for metabolizing plant phenolic acids to urolithins [10]; therefore, how to monitor the structure, concentration, and distribution of urolithins after catabolization by the gut microbial communities has become an attractive research topic for scientists. Metabolomics is the study of metabolites in a given biological system that provides correlation between specific metabolites and certain physiological conditions. For example, metabolomics has been employed for the screening of biomarkers for diagnosis, disease pathogenesis, and drug discovery [11–13]. Generally, the first step in a metabolomic analysis is to extract metabolites from the samples of interest. Those metabolites are often separated and analyzed using gas chromatography MS (GC/MS) and liquid chromatography MS (LC/MS) [6, 9]. Furthermore, the metabolites of interest could be identified through comparison of exact mass, retention time, and fragmentation information with genuine standards and spectral databases. LC-tandem MS spectrometry is a powerful tool for comparative metabolomic analysis. Using comparative metabolomic analysis would help us to understand the structural relationship of the target compounds and their derivatives. Therefore, using comparative metabolomics is an appropriate strategy for new urolithin derivatives discovery.

Urolithin A is a bioactive metabolite produced by the gut microbiota from ellagitannins and ellagic acid [7] (Figure 1). Recently, it has been proven that urolithin A prolongs lifespan of *C. elegans* and increases muscle function of mice [14]. In a male C57BL/6 mice study, it indicates that urolithin A presents in colon, intestine, liver, and prostate after receiving urolithin A by oral route [15]. Because urolithin A was detectable in mice prostate tissue, a human trial about patients with prostate cancer was reported. A double-blind and randomized trial was conducted in men with prostate cancer before radical prostatectomy [16]. The results suggest that urolithin A levels were inversely correlated with 8-OHdG expression in cancer tissues (*r* = −0.299, *P* = 0.017). Urolithin A and its related metabolites, urolithin B, and urolithin C have been found to possess the ability to inhibit the proliferation of human prostate cancer cells. However, it is not recommended to coadminister these compounds with the nonsteroidal antiandrogen drug bicalutamide [17]. In addition to prostate cancer, urolithin A also induces autophagy and inhibits metastasis in the human colorectal adenoma cell SW620 [18]. Other studies about colorectal cancer cells have been reviewed in the article [19], which suggests that some pharmacological mechanisms (apoptosis, autophagy, p21 expression, and G2/M arrest) are induced by urolithins.

In this study, we synthesized urolithin A and urolithin B with high purity by ourselves using the HPLC analysis. Then, we used the C57BL/6 mice model to identify the metabolites of urolithin A in urine by a single-dose urolithin A regimen. Furthermore, the mice were fed with ellagic acid, the urolithins' precursor, for continuously 14 days, and the plasma, liver, and feces samples were analyzed for ellagic acid and its urolithin metabolites. In addition to *in vivo* pharmacokinetic study, the cytotoxicity of urolithin A, urolithin B, and ellagic acid was also tested in four different human colorectal cancer cell lines.

## 2. Materials and Methods

### 2.1. Synthesis Method of Urolithin A and Urolithin B

#### 2.1.1. General Synthetic Procedure for Urolithin A

A mixture of 2-bromo-5-hydroxybenzoic acid (1.0 g), resorcinol (1.0 g), and sodium hydroxide (0.44 g) in water (3 mL) was heated under reflux for 30 min. After the addition of copper sulfate (10% aqueous solution, 0.5 mL), the mixture is refluxed again for 10 min when 3,8-dihydroxy-6H-benzo[c]chromen-6-one (urolithin A) was precipitated as a straw colored powder. The precipitate is filtered and washed with cold water. The residual solid was purified by column chromatography on silica (10% ethyl acetate/*n*-hexane), and the final purity of urolithin A was 98.1% by HPLC analysis.

3,8-Dihydroxy-6H-benzo[c]chromen-6-one (urolithin A): 0.57 g, yield: 54.0%, ^1^H NMR (500 MHz, DMSO-*d*_6_) *δ* 10.24 (br. s, 1H, 3-OH), 10.17 (br. s, 1H, 8-OH), 8.09 (d, *J* = 8.8 Hz, 1H, 5-H), 8.01 (d, *J* = 8.8 Hz, 1H, 6-H), 7.50 (d, *J* = 2.6 Hz, 1H, 9-H), 7.31 (dd, *J* = 2.6, 8.7 Hz, 1H, 7-H), 6.80 (dd, *J* = 2.3, 8.7 Hz, 1H, 4-H), 6.71 (d, *J* = 2.3 Hz, 1H, 2-H). ^13^C NMR (125 MHz, DMSO-*d*_6_) *δ* 160.62 (4C), 158.58 (4C), 156.98 (4C), 150.92 (4C), 126.97 (4C), 124.18 (3C), 123.82 (3C), 123.59 (3C), 120.19 (4C), 113.55 (3C), 113.05 (3C), 109.85 (4C), 102.87 (3C).

#### 2.1.2. General Synthetic Procedure for Urolithin B

A mixture of 2-bromobenzoic acid (5.5 g), resorcinol (5.5 g), and sodium hydroxide (2.2 g) in water (25 mL) was heated under reflux for 30 min. After the addition of copper sulfate (10% aqueous solution, 2.5 mL), the mixture is refluxed again for 10 min when 3-hydroxy-6H-benzo[c]chromen-6-one (urolithin B) was precipitated as a straw colored powder. The precipitate is filtered and washed with cold water. The residual solid was purified by column chromatography on silica (10% ethyl acetate/*n*-hexane), and the final purity of urolithin B was 99% by HPLC analysis.

3-Hydroxy-6H-benzo[c]chromen-6-one (urolithin B): 3.0 g, yield: 56.9%, ^1^H NMR (500 MHz, DMSO-*d*_6_) *δ* 10.35 (br. s, 1H, 3-OH), 8.25 (d, *J* = 8.1 Hz, 1H, 9-H), 8.18 (d, *J* = 7.9 Hz, 1H, 5-H), 8.15 (d, *J* = 8.8 Hz, 1H, 6-H), 7.88 (t, *J* = 7.5 Hz, 1H, 7-H), 7.56 (t, *J* = 7.5 Hz, 1H, 8-H), 6.85 (dd, *J* = 2.3, 8.8 Hz, 1H, 4-H), 6.75 (d, *J* = 2.3 Hz, 1H, 2-H). ^13^C NMR (125 MHz, DMSO-*d*_6_) *δ* 160.62 (4C), 159.87 (4C), 152.13 (4C), 135.31 (3C), 135.11 (4C), 129.68 (3C), 127.68 (3C), 124.85 (3C), 121.66 (3C), 118.96 (4C), 113.19 (3C), 109.39 (4C), 102.96 (3C).

### 2.2. Purification and Identification of Urolithin A and Urolithin B

The purity of the products was determined by HPLC, ^1^H-, and ^13^C NMR spectra. All chemical reagents in commercial quality were used as received (Sigma-Aldrich, St. Louis, MO, USA) and were used without further purification. Solvents were dried, and the synthesized compounds were purified using standard techniques. Reactions progression was monitored by TLC on aluminum plates coated with silica gel with a fluorescent indicator (Merck 60 F_254_). Unless otherwise stated. Melting points were determined in open capillaries using the Fargo MP-2D apparatus and are uncorrected. NMR spectra were recorded using TMS as an internal standard in DMSO-*d*_6_ at 500 MHz for ^1^H and at 125 MHz for ^13^C (Bruker Biospin GmbH AVANCE III 500 MHz, Rheinstetten, Germany). Chemical shift (*δ*) was reported in parts per million (ppm) measured relative to the internal standards tetramethylsilane (TMS), and the coupling constant (*J*) was expressed in Hertz (Hz). Column chromatography was performed with silica gel SiliaFlash® G60 (60–200 *μ*m) purchased from SiliCycle Inc. (Quebec City, Canada). In general, the reactions were carried out under anhydrous conditions in a dry solvent and nitrogen atmosphere. The purity of these compounds was based on the analysis of HPLC (Hitachi High-Technologies, Tokyo, Japan) equipped with a 280 nm detector and a LiChroCART RP-C_18_ column (4.6 mm i.d. × 250 mm, 5 *μ*m, Merck, Darmstadt, Germany). The mobile phase was composed of MeOH–H_2_O (0.05% TFA) (90 : 10), and the flow rate was 1.0 mL/min. The purity of all compounds was more than 98.1%. The mass spectra were acquired using a Thermo Finnigan model LXQ (Thermo Electron Co., Waltham, MA, USA) ion trap mass spectrometer equipped with ESI source interference and controlled by Xcalibur 2.06. The mass spectra were acquired in a positive ion mode or a negative ion mode [20].

### 2.3. Short-Term Urolithin A Metabolomics Experiment

Female C57BL/6 mice (*n* = 20, 8 weeks old, 16–19 g) were purchased from the National Laboratory Animal Center (Tainan, Taiwan). The diets and water were feed *ad libitum* and fasted 6 h prior to dosing. Mice were orally administered with urolithin A (0.6 mg/mouse, *n* = 10) or vehicle control (1.6% DMSO and 6.25% Tween-20 in 160 *µ*L water/mouse, *n* = 10). Urine samples were collected at 3, 6, 9, 12, and 24 h after oral administration. The samples were stored at −80°C until analysis. For the metabolomic analysis, the urine samples were thawed and 15 *μ*L was taken for extraction. Added 135 *μ*L methanol to each 15 *μ*L urine sample, vortexed and centrifuged at 13,200 g for 10 min at 4°C. The supernatants were collected and filtered by a 0.22 *μ*m filter for HPLC analysis.

### 2.4. Long-Term Ellagic Acid Metabolomics Experiment

Female C57BL/6 mice (*n* = 25, 18 weeks old, 19–24 g) were purchased from the National Laboratory Animal Center (Taiwan, Tainan). The diets and water were feed *ad libitum*. Mice were orally administered with low-dose ellagic acid (0.6 mg/mouse/day, *n* = 7), high-dose ellagic acid (1.2 mg/mouse/day, *n* = 10), or vehicle control (150 *µ*L water/mouse/day, *n* = 8). After administration of ellagic acid for 14 days, the mice were euthanized, and the plasma, liver, and feces were collected for further metabolomic analysis. In plasma samples, 15 *μ*L of each sample was mixed with 135 *μ*L methanol for extraction. The liver tissues were homogenized by an ultrasonicator for 5 sec, 4 times at 0°C in the extraction solvent (methanol: 12N HCl: water = 79.9 : 0.1 : 20, v/v/v), and the extraction volume (*µ*L): liver weight (mg) = 2 : 1. After ultrasonication, the samples were centrifuged at 13,200 g for 15 min at 4°C. For the fecal metabolite analysis, the cecum contents of mice were collected and then extracted using the method as used in liver tissue extraction. The supernatants were collected and filtered by a 0.22 *μ*m filter for HPLC analysis.

### 2.5. HPLC Analysis

All test samples were dissolved in methanol (HPLC grade) at a concentration of 10 mg/mL. The metabolite profile of each sample was analyzed using an HPLC system (SPD-M20A diode array detector, Shimadzu, Kyoto, Japan) coupled with a C18HQ column (4.6 mm i.d.  × 250 mm, 5 *μ*m, Interchim, Montluçon, France). The mobile phase consisted of 50% of methanol and 50% of ddH_2_O (0.05% of phosphoric acid), and the flow rate was 1 mL/min. In addition, urolithin A, urolithin B, and ellagic acid were quantified by HPLC with the same condition as aforementioned. For the calibration curve analysis, the stock solution was dissolved in methanol and then diluted to obtain the desired concentrations for the quantification (urolithin A and urolithin B: 200, 100, 50, 25, 12.5, and 6.25 *μ*M; ellagic acid: 4, 2, 1, 0.5, 0.25, and 0.125 *μ*M). The calibration curve was plotted using the linear regression method (peak areas versus compound concentrations).

### 2.6. UPLC-MS/MS Analysis

All test samples will be dissolved in methanol at a concentration of 10 mg/mL. The experimental procedure was conducted as previously described [7]. The mass data were acquired using UPLC-ESIMS (Thermo Scientific Orbitrap Elite Mass Spectrometer), and the mass range was set up as m/z 100–1500.

### 2.7. Human Colorectal Adenocarcinoma Cell Lines

There were 4 cell lines used in this study including SW480, SW620, HCT 116, and HT-29 cells (Bioresource Collection and Research Center, Hsinchu, Taiwan). SW480 and SW620 cells were established from a same Caucasian male patient with colon adenocarcinoma, and SW480 was isolated from the primary colon tumor site, SW620 was isolated from a lymph node metastasis site. SW480, but not HT-29, secrets granulocyte-macrophage colony-stimulating factor (GM-CSF) that stimulates the proliferation of acute megakaryoblastic leukemia M-07e [21]. SW480 and SW620 synthesize small quantities of carcinoembryonic antigen (CEA) and belong to Broders' grade 4 cancer [22]. By Northern blot analysis, the oncogenes c‐myc, H‐ras, K‐ras, N‐ras, myb, fos, and p53 were all expressed in SW480 and SW620 cells [23]. HT-29 cells were isolated from a Caucasian female patient with colorectal adenocarcinoma (ATCC data). HT-29 and SW480, but not SW620, express vitamin D receptor whose activation by 1,25-dihydroxyvitamin D inhibits cell proliferation and clonogenic growth in soft agar [24]. HT 116 has a mutation in codon 13 of the ras protooncogene and expresses transforming growth factor beta 1 and beta 2. SW480 and SW620 cells are cultured in Leibovitz's L-15 medium with 10% FBS, and HT-29 and HCT 116 cells are cultured in McCoy's 5a medium with 10% FBS.

### 2.8. Cytotoxic Analysis of Urolithin A, Urolithin B, and Ellagic Acid in Cells

The cytotoxicity of urolithin A, urolithin B, and ellagic acid was analyzed in these 4 cell lines by 3-(4,5-dimethylthiazol-2-yl)-2,5-diphenyltetrazolium bromide (MTT) assay, following the method outlined in a previous study [25]. Cells (6.5 × 10^4^ for SW480, 1.5 × 10^5^ for SW620, 2.5 × 10^5^ for HCT 116, and 1 × 10^5^ for HT-29) were cultured in 24-well plates for 24 h and then were incubated with various concentrations of chemicals for another 24 h. After adding MTT to the medium and incubating the cells for 1.5 h (SW480 and SW620) and 45 min (HCT 116 and HT-29), the cells were dissolved in DMSO and the suspension was collected for OD570 detection by a spectrophotometer. The result was expressed as a percentage, relative to control group.

### 2.9. Statistical Analysis

The values shown are the mean ± SE. Data were statistically evaluated by GraphPad Prism software (version 9.4) using student's *t*-test for comparison of the two groups, and for more than two groups, we conducted by ANOVA with Dunnett's multiple comparisons test; significant differences are shown as ^*∗*^*p* < 0.05, ^*∗∗*^*p* < 0.01, and ^*∗∗∗*^*p* < 0.001.

## 3. Results

### 3.1. Synthesis of Urolithin A and Urolithin B

**Scheme 1:**
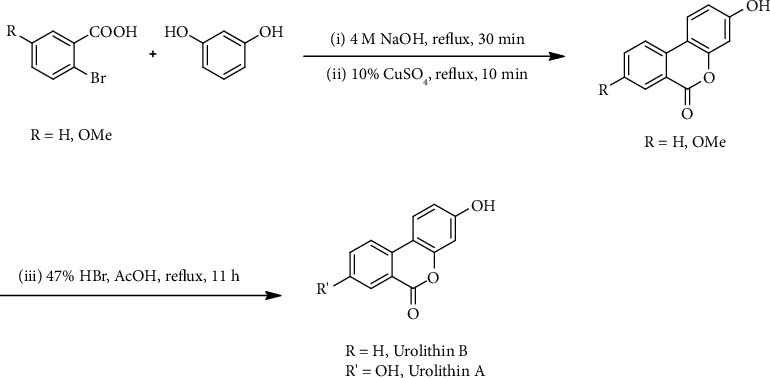
Synthesis of urolithin A and urolithin B.

As shown in Scheme 1, a mixture of 2-bromo-5-hydroxybenzoic acid or 2-bromobenzoic acid, resorcinol, and sodium hydroxide in water was heated under reflux for 30 min. After the addition of copper sulfate (10% aqueous solution), the mixture is refluxed again for 10 min. 3,8-Dihydroxy-6H-benzo[c]chromen-6-one (urolithin A) or 8-dihydroxy-6H-benzo[c]chromen-6-one (urolithin B) was precipitated, filtered, washed with ice-water, and crystallized out after 6 h obtained as a light white-yellow powder, and the final purity of urolithin A and urolithin B was 98.1% and 99% by HPLC analysis [20].

### 3.2. Purification and Identification of Urolithin A and Urolithin B

Urolithin A and urolithin B have been synthesized from 2-bromo-5-hydroxy benzoic acid or 2-bromo-benzoic acid in two steps as described in Section 1.1. The purified urolithin A and urolithin B were obtained as a slight yellow color solid, which was characterized using NMR spectroscopy and HPLC analysis. The purity of these compounds was based on the analysis of HPLC (Hitachi High-Technologies, Tokyo, Japan) equipped with a 305 nm detector and LiChroCART RP-C_18_ column (4.6 mm i.d. × 250 mm, 5 *μ*m, Merck, Darmstadt, Germany). The mobile phase was composed of MeOH–H_2_O (0.05% phosphoric acid) (50 : 50), and the flow rate was 1.0 mL/min. The purity of all compounds was greater than 98.1%. Urolithin A and urolithin B showed retention time of 12.3 min and 22.3 min, respectively.

### 3.3. Short-Term Metabolomics Analysis of Urolithin A Intake in Mice

**Figure 2:**
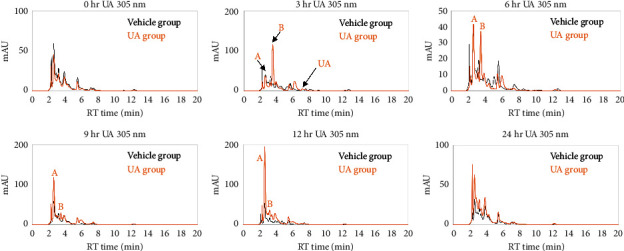
HPLC analysis of mice urine samples of vehicle control (1.6% DMSO and 6.25% Tween-20 in 160 *µ*L water, black line) and urolithin A (0.6 mg/mouse, orange line) group. The urines were collected at indicated time points (0, 3, 6, 9, 12, and 24 h) after gavage feeding of urolithin A. The retention time (RT) of urolithin A and urolithin B is 7.6 min and 15.5 min, respectively.

The short-term metabolomics analysis using the urine sample collected from C57BL/6 mice fed with urolithin A (0.6 mg/mouse) or vehicle control (1.6% DMSO and 6.25% Tween-20 in 160 *µ*L water/mouse). The urine samples were collected after gavage with vehicle/urolithin A for 0, 3, 6, 9, 12, and 24 h and then subjected to the HPLC analysis. The results showed that urolithin A (RT = 7.6 min) was only detectable at 3 h, it suggested that urolithin A was rapidly metabolized before 3 h, and some unknown materials were detected by HPLC analysis as well (Figure 2). The unknown materials included the signal **A** (RT = 3.0 min) which was detected in urine samples of mice after feeding with urolithin A for 6 h to 24 h and another signal **B** (RT = 3.6 min) which was detected in urine samples of mice after feeding with urolithin A for 3 h and 6 h. Because the 2 unknown materials were more hydrophilic than urolithin A, it is suggested that they might be urolithin A-related metabolites by mice phase II metabolic enzymes. However, the exact structure of those metabolites remains unknown, and further UPLC-MS/MS analysis for structural elucidation was applied.

### 3.4. UPLC-MS/MS Analysis

**Figure 3:**
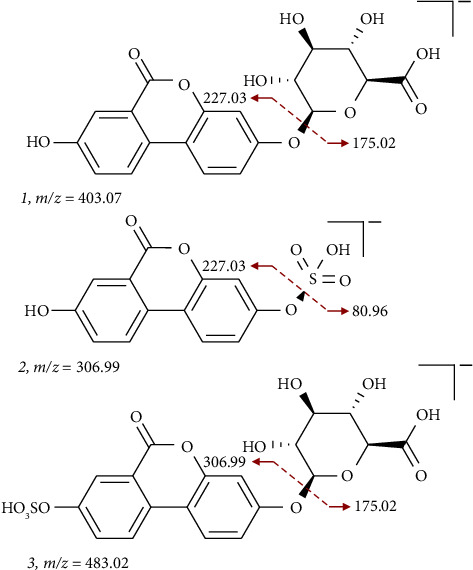
Proposed structures of urolithin A-related metabolites using UPLC-MS/MS.

To check the possible urolithin A-related metabolites in the urine samples, the UPLC-MS/MS analysis was performed in this study. Because the urine samples were limited, we used pooling sample for this assay. Here, we detected and proposed three possible urolithin A-related metabolites, including urolithin A 3-O-glucuronide (**1**) was detected at m/z = 403.07 and the MS^2^ ion at 227.03 (characteristic for neutral loss of 176 amu); urolithin A 3-sulfate (**2**) was detected at m/z = 306.99 and the MS^2^ ion at 227.03 (characteristic for neutral sulfate loss of 80 amu), and urolithin A-sulfate glucuronide was detected at m/z = 483.02 and the MS^2^ ion at 306.99 (neutral loss of 176 amu), which fragmented to 227.033 (characteristic for neutral sulfate loss of 80 amu) (**3**) in the urine samples (Figure 3). Based on aforementioned, we speculated that the unknown signals **A** and **B** of urine samples in Figure 2 may be phase II conjugates of urolithin A.

### 3.5. Long-Term Metabolomics Analysis of Ellagic Acid Intake in Mice

**Figure 4:**
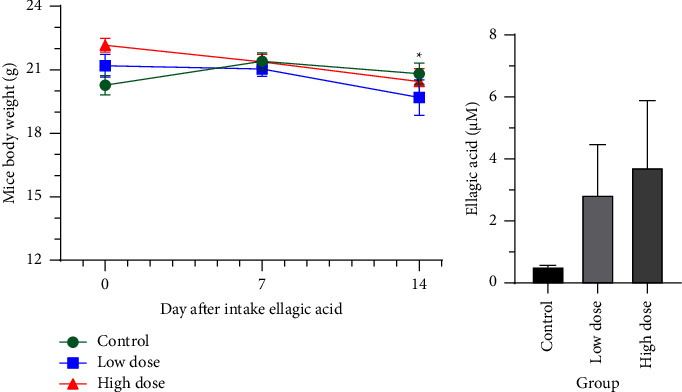
(a) The mice body weight change after taking ellagic acid or vehicle. The mice were fed low-dose ellagic acid (0.6 mg/mouse/day), high dose (1.2 mg/mouse/day), or vehicle from day 1 to day 14. ^*∗*^*p* < 0.05 compared to day 0 of each group. (b) Ellagic acid concentration of fecal extraction samples in mice (*n* = 5-6/group).

After feeding mice with ellagic acid continuously for 2 weeks, the mice were euthanized and the feces, liver, and plasma of mice were collected and stored in −80 °C freezer for further analysis. The mice body weight change is shown in Figure 4(a), and it showed that high-dose ellagic acid intake (1.2 mg/mouse/day) reduced the mice body weight after continuous intake for 14 days. For the plasma and liver samples, no ellagic acid and urolithin A-related signals were detected in HPLC analysis. We also conducted the fecal metabolites extraction and the HPLC analysis. It showed that the ellagic acid (RT = 5 min) contents could be detected in the fecal samples in both of the low- and high-dosage groups, while the control group showed a very low ellagic acid concentration compared to the other two groups (Figure 4(b)). In addition, the ellagic acid contents showed a dose-dependent manner in low (2.8 *μ*M) and high-dosage groups (3.7 *μ*M). However, urolithin A was unable to detect in fecal samples in both of low- and high-dosage groups (data not shown).

### 3.6. Cytotoxic Analysis of Urolithin A in Cells

**Figure 5:**
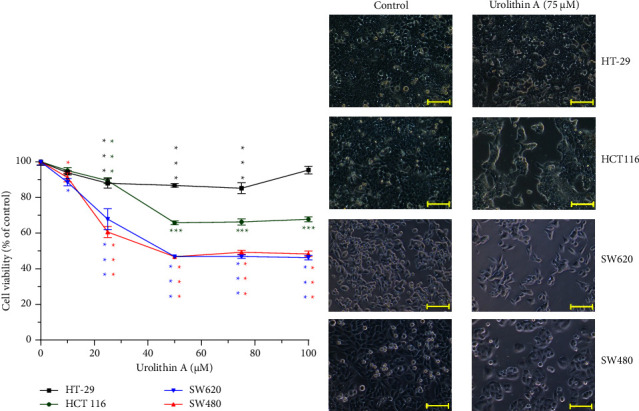
Cytotoxicity of urolithin A in HT-29, HCT 116, SW480, and SW620 cells. (a) Each cell viability was compared with its own control. ^*∗*^*p* < 0.05 and ^*∗∗∗*^*p* < 0.001. (b) The photographs show cells that were treated with or without 75 *µ*M urolithin A for 24 h. The yellow scale bar is 100 *µ*m.

Urolithin A was dissolved in DMSO, and the stock concentration was 100 mM. In a dose-dependent cytotoxic assay, the final concentration of DMSO in the culture medium was 0.1%. As shown in Figure 5, IC_50_ of urolithin A in SW480 and SW620 was about 50 *μ*M which had reached the plateau with 50% cell viability. In HCT 116, the cytotoxicity reached the plateau at 50 *μ*M with about 75% cell viability. In HT-29 cells, the cell viability was more than 80% by 20–100 *μ*M urolithin A treatment for 24 h, and it means urolithin A had minor cytotoxicity in HT-29 cells. In summary, urolithin A has different cytotoxicity in SW480, SW620, HCT 116, and HT-29 cells, but all reached the plateau at 50 *μ*M.

### 3.7. Cytotoxic Analysis of Urolithin B in Cells

**Figure 6:**
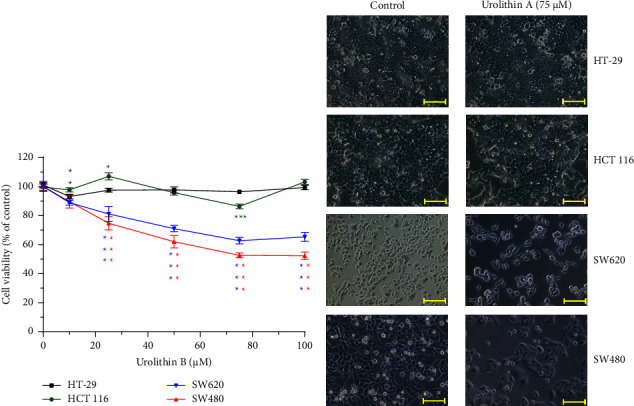
Cytotoxicity of urolithin B in HT-29, HCT 116, SW480, and SW620 cells. (a) Each cell viability was compared with its own control. ^*∗*^*p* < 0.05, ^*∗∗*^*p* < 0.01, and ^*∗∗∗*^*p* < 0.001. (b) The photographs show cells that were treated with or without 75 *µ*M urolithin B for 24 h. The yellow scale bar is 100 *µ*m.

We also analyzed the cytotoxicity of urolithin B in cells. As shown in Figure 6, IC_50_ of urolithin B in SW480 was about 75 *μ*M, which was also the highest cytotoxicity with 50% cell viability. In SW620 cells, the cytotoxicity reached the plateau at 75 *μ*M with about 60% cell viability. There was no obvious cytotoxicity of urolithin B in HT-29 and HCT 116 cells. In conclusion, the cytotoxicity of urolithin B was lower than that of urolithin A in these 4 cell lines.

### 3.8. Cytotoxic Analysis of Ellagic Acid in Cells

**Figure 7:**
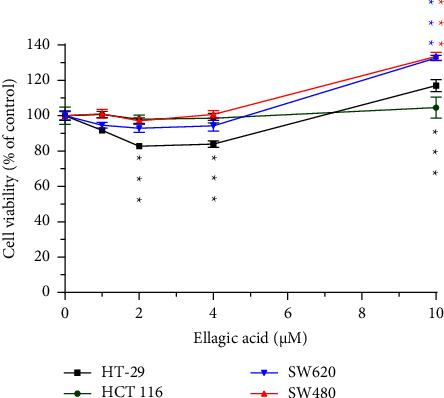
Effect of ellagic acid on cell viability in HT-29, HCT 116, SW480, and SW620 cells. Each cell viability was compared with its own control. ^*∗∗∗*^*p* < 0.001.

In 1–4 *μ*M of ellagic acid, the cell viability was not below 80%, and 10 *μ*M ellagic acid increased cell proliferation in HT-29, SW620, and SW480 cells (Figure 7). It suggests no obvious cytotoxicity of ellagic acid in these 4 colorectal cancer cell lines, and it instead to increase cell proliferation at 10 *μ*M. In addition, we used transformed normal epithelial cells SV-HUC1 for the cytotoxic assay, and they also showed no cytotoxicity of ellagic acid. For the cytotoxicity of urolithin A and urolithin B, they exhibit a similar effect to SW620 cells in SV-HUC1 (data not shown).

## 4. Discussion

In 2003, urolithins were first revealed as bioavailable metabolites from the ellagitannins of pomegranate in rat [26, 27]. The ellagitannins are hydrolysable tannins that are found in many medicinal plants, herbal teas, and foods [28]. However, the low bioavailability of ellagitannins limited their health benefits. The gut microbiota metabolizes ellagitannins and ellagic acid to several urolithins that were reported to have various health effects in animal models and humans [28]. The characteristic chemical structure of urolithins, including urolithin A, urolithin B, urolithin C, isourolithin A, urolithin M7R, urolithin CR, and urolithin AR, is an *α*-benzocoumarin scaffold [10]. The urolithins are transformed from ellagic acid by the gut microbiota through lactone-ring cleavage, decarboxylation, and dihydroxylation [28]. In addition, the major urolithins found in plasma and tissues and excreted in urine and feces are urolithin A, urolithin B, and isourolithin A [8]. The corresponding phase II conjugates (glucuronides or sulfates) of urolithin A have also been found in the aforementioned urine samples as well [10].

The high variation of urolithins concentration in biological fluids was reported. For example, the concentration of urolithin A reported in human (consuming nectar 20 g/day for 3 weeks) urine is about 4807 nM, while the concentration of urolithin B in the same sample is about 312 nM [29].  Wistar rats were fed a diet containing strawberries that were rich in monomeric ellagitannins (ME), dimeric ellagitannins (DE), or free ellagic acid (FE) at a concentration of 0.10-0.15% for 24 h. The urine concentrations of urolithin A in the ME, DE, and FE groups were found to be 5.6, 3.9, and 99.5 μM, respectively [30]. In the same model, the concentration of urolithin A in the ME, DE, and FE groups is 8.3, 0.2, and 224.3 μg/g of cecal digesta, respectively. However, urolithin A was only detected in the feces sample of the FE group (89.3 μg/g feces) [30]. The variation may be due to the sample dosage, the source of ellagitannins, and the methods used for quantification. Our results revealed that 3 possible phase II conjugates (glucuronides or sulfates) of urolithin A were detected in the urine samples after feeding mice with 0.6 mg urolithin A. The results are consistent with previous studies. After absorption by animals, only traces of nonconjugated urolithins are able to detect in bodily fluids, indicating that urolithins undergo phase II metabolism in a very short time. This also affects the presence of urolithins in plasma and tissues. The presented urolithins predominant in conjugated form included glucuronides (major) and sulfates (minor) [31].

In a cytotoxicity assay of SW480, SW620, HCT 116, and HT-29 cells, it shows that there is no obvious cytotoxicity in ellagic acid-treated cells; in contrast, a high-dose (10 *μ*M) ellagic acid induced cell proliferation (Figure 7). In split of the antiproliferation effect of ellagic acid in the breast cancer cell line MCF-7 [32], we still suggest that foods with high ellagic acid content should be consumed with caution by CRC patients. In contrast, the metabolites of ellagic acid, urolithin A and urolithin B, have cytotoxicity to SW480, SW620, and HCT 116 (Figures 5 and 6), but it is necessary to convert ellagic acid to urolithins by intestinal microbiota. In a human study using oral pomegranate extracts, the urolithin A and urolithin B are presented in normal and malignant tissue of CRC patients [7], it suggests that pomegranate extracts could be metabolized to urolithins in the human intestine. In this study, urolithin A-related compounds could not be detected in mice plasma, liver, and feces after ellagic acid intake for 14 days, but ellagic acid was measured in the feces. This mice study shows different results with the above human study. Some possible explanations for the differences between the two studies: one is the intake material; the human study used pomegranate extracts, and we used ellagic acid. Second one is a human study, and we did a mouse experiment. Third, the assay tissues are different. The human study analyzed colon tissues, and we investigated plasma, liver, and feces. In addition, the differences in gut microbial ecology influence the variability in microbial metabolism of ellagic acid; for example, phenotype 0 does not produce any urolithin [6]. Neither urolithin A nor urolithin B reduced cell viability to less than 40% (Figures 5 and 6). Especially in HT-29 cells, urolithin A produced lower cytotoxic activity than in SW480, SW620, and HCT 116 cells. One report provides that the resistance mechanism of HT-29 to urolithin A is phase II metabolism which converts urolithin A to urolithin A-glucuronide which has lower cytotoxic activity [33, 34]. In this study, we found that urolithin A was rapidly metabolized and excreted into urine in mice (Figure 2); it suggests that the single dose of urolithin A might exert a limited biological effect in vivo. In a previous study in mice [15], it shows that after urolithin A intake, little urolithin A was detected in mice plasma and high concentration of urolithin A presented in the prostate, intestine, and colon, and high concentrations of urolithin A-sulfate and urolithin A-glucuronide presented in the liver and kidney. Therefore, our data support the abovementioned report because minor amounts of urolithin A presented in mice urine that reflect its low concentration in mice plasma. The urolithin A-related metabolites were detected after intake for 3 h to 24 h (Figure 2). The phase II metabolites of urolithin A, including urolithin A-glucuronide and urolithin A-sulfate, also show no effect on CRC cell proliferation and senescence [35]. Therefore, if urolithin A could directly enter colon epithelial cells without phase II metabolism, it produces its own biological activity. If urolithin A is absorbed into blood, it might be rapidly metabolized by liver phase II enzymes and produce the metabolites with lower biological activity.

Among the urolithins, urolithin A has been extensively studied for its various biological activities, which include anti-inflammatory properties, neuroprotective effects, cognitive improvement, and cardioprotective effects [9]. In this study, we also found that urolithin A was better than urolithin B and ellagic acid in anti-CRC cell proliferation. However, urolithin A is a compound derived from the gut bacteria that can be naturally converted from dietary precursors to meaningful levels in only 40% of people. In several recent studies, the positive effects of direct urolithin A administration on health, aging, and age-related conditions have been identified [36–38]. Urolithin A is obtained through pharmaceutical synthesis as a modern dietary supplement. Therefore, using urolithin A directly might be a more effective route than ellagitannins or ellagic acid intake in cancer inhibition of CRC patients. A human study also suggests that direct urolithin A supplementation could overcome the limitation of gut microbiome variability to produce a consistent effect [39].

## 5. Conclusions

In this study, we developed the method for the synthesis, purification, and identification of urolithin A and urolithin B. In mice study, it suggests that urolithin A could be absorbed into blood and might be metabolized rapidly by phase II enzymes. In cell cytotoxicity assay, urolithin A has higher cytotoxicity than urolithin B and ellagic acid, and HT-29 cells have resistance to urolithin A compared to HCT 116, SW480, and SW620 cells. Due to the enhanced proliferation of CRC cells by ellagic acid, it is preferable to directly use urolithin A instead of natural extracts containing ellagitannins and ellagic acid in patients with colorectal cancer.

## Data Availability

The datasets used in the current study are available from the corresponding author on reasonable request.
